# Correspondence

**Published:** 1901-03

**Authors:** N. H. Reeve

**Affiliations:** Secretary


Bristol, Tenn.-Va.,
February 18, 1901.

An organization has been effected to establish a home for destitute orphans of deceased members of our profession.

This movement, beginning as it does with the new century, adds still greater interest to the enterprise. The name "Physicians' Orphans' Home" is self-explanatory, and appeals at once to the benevolence of every member of our noble fraternity, a plan which, in its conception and development, embodies a genuine *charity*. The plan contemplates the establishment of a home designed for the maintenance and education of the helpless little ones of our dead, which should at once awaken the philanthropic spirit which pervades the breast of every man who has a soul, and prompt a quick response to a call for aid in this grand enterprise, thereby erecting a lasting monument to the memory of those who witnessed the death of the old century and the birth of the new. The plan is to establish and maintain the home by endowments and subscriptions from the profession, and will be under the management and control of the regular medical profession. There are in the United States approximately one hundred thousand regular physicians, represented mainly in the American Medical Association, and in the various state, tri-state, city and county societies. An average subscription of $5.00 each would make a total of $500,000.00, one-half of which would equip and maintain for a number of years an orphanage of considerable proportions. The Committee on Location, after considering Asheville, N. C., Louisville, Ky., Colorado Springs, Col., Canton, Ohio, and Lookout Mountain, Tenn., unanimously elected Bristol, Tenn.-Va., by reason of its high altitude and southerly mountain climate, first, and because Bristol is centrally located with refereuce to the physicians of the United States. Then, too, the liberality shown the Committee through the Board of Trade, in the way of a handsome bonus in a magnificent building of 85 furnished rooms, and grounds situated so that additions can be made at any time necessary. There remains but $35,000.00 to be paid on this property, which is but little more than one-third of its intrinsic value. The inducement to purchase this handsome property was overwhelming, since there were no objectionable features existing to this ideal location. A full description of this property, with pictures of the building and all necessary literature, will be mailed to every regular physician in the United States. Correspondence solicited.

N. H. REEVE, M.D., Secretary.

